# The Prevalence of Autoimmune Disorders in Women: A Narrative Review

**DOI:** 10.7759/cureus.8094

**Published:** 2020-05-13

**Authors:** Fariha Angum, Tahir Khan, Jasndeep Kaler, Lena Siddiqui, Azhar Hussain

**Affiliations:** 1 Internal Medicine, Xavier University School of Medicine, Oranjestad, ABW; 2 Medicine, Xavier University School of Medicine, Oranjestad, ABW; 3 Biology, Long Island University Post, New York, USA; 4 Healthcare Administration, Franklin University, Columbus, USA

**Keywords:** autoimmune disorders, lupus, sex chromosomes, systemic lupus erythema

## Abstract

Autoimmune disorders are characterized as a condition in which the host's immune system mistakenly attacks itself. These disorders cause the immune system to cause a systemic reaction by attacking multiple organs or may be localized to attacking one specific organ, such as the skin. The exact mechanism of such autoimmune conditions is not well understood; however, the presumed mechanism tends to vary amongst the disorders. Autoimmune diseases present with a clear gender bias with a greater prevalence amongst women, occurring at a rate of 2 to 1. Many autoimmune disorders tend to affect women during periods of extensive stress, such as pregnancy, or during a great hormonal change. A far greater number of women are affected every year with autoimmune diseases, leading to researchers attempting to identify the underlying factors, which could be responsible for this disparity. Autoimmune disorders occur as a result of multiple factors as some disorders may be genetic, while others are sporadic. Throughout this review, various hypotheses are explored that provide insight into the increased susceptibility of autoimmune disorders within women.

## Introduction and background

Autoimmune disorders are conditions in which the immune system is unable to differentiate between healthy tissue and potentially harmful antigens. The immune system attacking its own host can be explained through the concept of molecular mimicry. In a normal case, the immune system will attack the foreign antigens and produce a response with respect to the antigens. In the case of autoimmune disorders, the immune system is unable to differentiate from foreign antigens and its own host cells. Molecular mimicry is known as a mechanism in which a foreign antigen holds structural similarities as self-antigens. Although the research around its association with autoimmune conditions, molecular mimicry remains a key mechanism that might be involved in the initiation of autoimmunity. Molecular mimicry causes self-destructing attacks that can cause a plethora of reactions to manifest within the body ranging from minor to life-threatening. The presentation of various autoimmune conditions differs, along with the age of onset. 

Table 1 is a tabular presentation of the various autoimmune conditions discussed, along with the average age of onset. The onset of Sjogren’s syndrome is typically seen around the ages of 40-60; however, mild signs are often overlooked, leading to a delayed diagnosis. The onset of SLE can be seen between the ages of 15 and 55 years; often individuals diagnosed earlier on in life tend to have a more severe form of SLE. Systemic sclerosis is usually diagnosed between 20 and 50 years of age. Rheumatoid arthritis is diagnosed between the ages of 30 and 60 years, while psoriasis is diagnosed between 15 and 35 years of age. These are a small minority of the vast amount of autoimmune diseases that affect 20% of the entirety of the human population. There are over 100 types of autoimmune diseases that predominantly affect women. Approximately 80% of all patients diagnosed with autoimmune diseases are women [1]. Sjogren’s syndrome, an autoimmune disease characterized by chronic dry eyes and mouth due to the degeneration of lachrymal and salivary glands, affects women in a 9:1 ratio [2]. SLE, an autoimmune disease in which the body attacks healthy tissues affecting the skin, joints, kidneys, and the brain, are seen to affect women in a 7:1 ratio [3]. Rheumatoid arthritis, a chronic inflammatory joint autoimmune disease that can immobilize fingers, wrists, feet, and ankle joints, affects women in a 3:1 ratio [4]. Systemic sclerosis, an autoimmune disease affecting the skin and internal organs of patients due to a collagen defect, affects women in a 3:1 ratio [5]. As the review reflects, women tend to develop autoimmune diseases more often than men throughout the course of their lifetime. The exact etiology of autoimmune disorders is said to be unknown; however, it has been postulated that it may be multifactorial. Researches have also postulated the association of autoimmune conditions with the X chromosome and X inactivation. A female individual normally has two X chromosomes, and for this reason, possesses a higher risk of autoimmune diseases, as compared to men. Recent researches address the possible cause for the differences in male and female immune systems. These differences address the reason as to why women are more susceptible to autoimmune diseases compared to men, as this review will aim to explore.

**Table 1:** Autoimmune disorders and the average age of onset SLE, systemic lupus erythematosus

Autoimmune condition	Average age of onset
SLE	15-55
Systemic sclerosis	20-50
Rheumatoid arthritis	30-60
Psoriasis	15-35
Sjogren’s syndrome	40-60

## Review

Sex chromosomes

Men and women alike are born with 23 pairs of chromosomes, with 22 pairs of autosomes and one pair of sex chromosomes, the differentiating factor between the two genders. Women have XX sex chromosomes, while men have XY sex chromosomes. The X and Y sex chromosomes vary greatly in size as the X chromosome is physically larger than the Y chromosome. This disproportion between the two chromosomes suggests that the X chromosome contains more genes than the Y chromosome [6]. The X chromosomes contain approximately 800-900 genes coded to provide instructions for proteins, entailing for 5% of total DNA in human cells, whereas, the Y chromosomes contain approximately 50-60 genes coded to provide instructions for proteins and this entails about 2% of total DNA in human cells [7,8]. Furthermore, the X chromosome also stains for a greater amount of immune-related genes as well as immune regulatory genes, which aids and induces immunological responses in the body [9]. The larger number of genes originating from the X chromosome creates a far greater possibility of a larger number of mutations occurring. This puts women at a greater risk for the development of autoimmune diseases solely due to women having two X chromosomes, whereas men possess only one. The presence of two X chromosomes essentially creates a 'double dose' of genes present on the X chromosome and because of this, predisposes the female to autoimmunity. 

**Figure 1:**
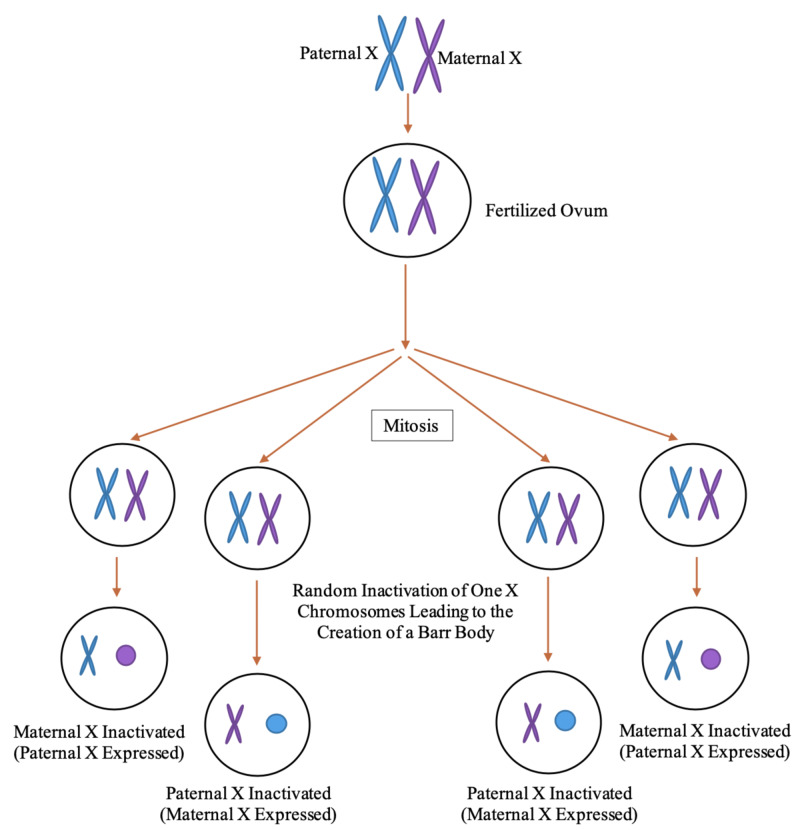
Process of X inactivation creating Barr bodies

During the early stages of embryonic development, females undergo a phenomenon known as X inactivation, as depicted in Figure 1. X inactivation occurs in order to prevent the overexpression of genes as genes present on one of the two X chromosomes, in each cell, are silenced. In certain cells, the X chromosome inherited from the mother is inactivated, while in other cells the X chromosome inherited from the father is inactivated [10]. This highlights that, genetically, all women are mosaics and will genetically vary from cell to cell regarding their X chromosomes [11]. Some may be genetically more skewed toward their maternal X chromosome, while others are more skewed toward their paternal X chromosome, due to X inactivation being a randomized process on a cell-to-cell basis [9,11]. Those skewed more so toward either their maternal or paternal X chromosome may not be able to recognize the opposing X chromosome causing the rise in polymorphic antigens. Despite originating from the body, polymorphic antigens are viewed as a foreign substance invading the body and will elicit an immune response. The immune response would result in the production of antibodies against the assumed foreign substance. This will then bring forth an immune response, ultimately causing the body to attack itself unknowingly due to a genetic variation [12]. Currently, this is one of the leading theories for the cause of SLE. 

Systemic lupus erythematosus

With a predominance of 7:1 in women, SLE is thought to be due to an overexpression of CD40LG and CXCR3 genes that result from the lack of X inactivation. A patient diagnosed with SLE experiences debilitating fatigue, fever, pain, and the characteristic butterfly rash seen on the face. Research conducted upon mice and humans diagnosed with SLE at the School of Veterinary Medicine at the University of Pennsylvania may lead to vital insight [12]. This experiment focused predominately upon the T cells of mice and SLE patients. T-cells are a specific type of white blood cells that are imperative to the immune system, specifically adaptive immunity. Adaptive immunity controls the body’s immunity against new and previously exposed pathogens. During this experiment, a single mouse cell, as well as an SLE patient cell were both isolated in order to delve into the epigenetic features of X inactivation three different types of cells: the T-cells, mature T-cell subsets, and developing thymocytes. The experiment concluded that X chromosome inactivation is key in preventing the overexpression of certain genes. These genes play an essential role in immunity, as many encode for proteins such as antibodies, and thus, an overexpression will result in an overproduction of antibodies.

X chromosome inactivation takes place during the embryological stage of female development in order for the biochemical uniformity of the cells. Much of the inactivation is controlled by a particular upregulated allele of the long noncoding RNA X-inactive specific transcript (Xist) from the soon to be inactive X [13]. This Xist RNA will then continue to place multiple heterochromatin modifications (H3K27me3 & H2a-ubiquitin) upon a given X chromosome in order to prevent transcription of the chromosome, rendering it as inactive. As the development of the female embryo continues to occur, the cells continuously divide and as the cells divide, the inactivated X will remain as such. The heterochromatin is vital in this process as it acts to suppress genes, which are necessary for the inactivation of the X chromosome. It was found throughout the experiment that the cells of female humans and mice alike did not have the inactivation of the designated inactivated X chromosome in mature T- and B-cells. Furthermore, the Xist RNA and heterochromatin H3K27me3, which are necessary for X inactivation, were not present during T-cell differentiation in the thymus. This resulted in mature impaired T-cells, which lacked X chromosome inactivation. 

An adequate T-cell function is vital as it is responsible for recognizing and neutralizing foreign viruses and bacteria in the body. When there is an influx of T-cells without a virus or foreign bacteria present, the cells will grow restless and begin to attack the host [14]. This gives rise to an autoimmune response where the body’s defense mechanism against foreign antigens is compromised. The overexpression of immune regulatory genes from the lack of X chromosome inactivation will create autoantibodies against the cells of the body, and ultimately weaken the body’s immune system and damage vital organs leaving the body defenseless against true foreign antigens. SLE is one example of the many different autoimmune disorders affecting women more. Currently, the lack of X chromosome inactivation, causing increased X chromosome activity, is one of the leading theories for the cause for SLE, especially in women. Each autoimmune disorder varies in regard to the age of onset, symptoms, and overall effect upon those diagnosed.

VGLL3 transcription factor

The female predilection of autoimmune disorders follows a bimodal curve as these disorders are more commonly seen in the embryological period or post-menopausal. Evolutionarily, females have a greater amount of VGLL3, which is a unique transcription factor that regulates genes and has a firm association with autoimmune diseases such as Sjogren’s syndrome, SLE, and scleroderma [15]. VGLL3 elicits a cutaneous inflammatory response in SLE patients such as a butterfly skin rash across their cheeks and nose, by upregulating inflammatory genes [16]. In the following experiment conducted in which the VGLL3 transcription factor of humans was reviewed under high-resolution global transcription analyses regarding its relation to autoimmune disorders. The experiment analyzed skin biopsies of 31 women and 51 men via whole-genome RNA sequencing. Essentially, 661 core genetic variations related to immunity were found between women and men, with an upregulation of immune genes in women specifically near the loci of SLE and systemic sclerosis [15]. Thereby, it was evident that there was a female-biased molecular signature that coincides with increased female susceptibility to autoimmune diseases [14,15]. This molecular signature directly correlates with having two X chromosomes, like in females, causing an upregulation of the VGLL3 gene [15]. 

In a separate experiment also conducted on women and female mice, it was observed that compared to men and male mice, female mice and women had increased levels of VGLL3 in their epidermis. In fact, female mice contained 2.8-fold greater levels of VGLL3 expression than male mice. Furthermore, transgenic mice that expressed increased levels of VGLL3 were generated in order to see if SLE-like symptoms would appear in the mice. Although at birth transgenic and control pups appeared identical, by 6-12 weeks, those with increased levels of VGLL3 displayed progressive scaling and skin thickening around the same areas humans with SLE would normally display, such as the ears and face. Cutaneous inflammation could also be seen in the transgenic mice, and further experimentation upon their cells revealed increased production of T- and B-cells which caused increased lymphocyte expansion, increased autoantibodies of the B cells along with further tissue damage, as seen in SLE patients [17]. Thereby demonstrating a direct correlation between increased levels of VGLL3 playing a great role in the causality of SLE.

VGLL3 is present in the epidermis of both men and women. The epidermis is a prime location for VGLL3 as it is the largest organ of the body, a sensitive indicator of immune dysfunction, and the first line of defense against antigens. VGLL3 has a strong correlation to SLE, which presents with characteristics features such as a butterfly rash, discoid rash, photosensitivity, and mucosal ulcers [15]. It is unclear as to why women contain more VGLL3 in their epidermis, although it has been speculated that it may be due to an evolutionary adaption to help develop a stronger immune system and ward off infection, however, at a great cost [15,18]. Due to the increased amount of VGLL3 present on the epidermis through evolutionary adaption, it has left women with an increased autoimmune response, ultimately causing harmful and often life-threatening autoimmune disorders to manifest [18]. Thus, via a multitude of factors exclusive to those with two X chromosomes, such as its ability to code for a greater amount of genes due to larger size, X inactivation, as well as an increased amount of transcription factors present in skin, all contribute to crucial genetic biological difference between men and women leading to increased susceptibility of autoimmune diseases in women.

Psoriasis

Psoriasis is an autoimmune disorder characterized by the increased thickness of the stratum corneum layer. This hyperkeratosis leads to the formation of red, salmon, and white-colored scaly, itchy plaques. These plaques can occur anywhere on the skin but predominantly seen on the back, elbows, knees, and scalp. This debilitating disorder can often cause a great deal of physical, mental, and emotional stress upon those diagnosed. With a prevalence of 11% in Caucasian and Scandinavian populations, psoriasis is generally impacted 2% of the global population. However, in African and Asian populations, less than 1% of individuals are affected. Often triggers such as trauma, stress, colder climates, smoking, alcohol, and infections can increase flare-up frequency as well as worsen the symptoms [19]. Typically, the severity of psoriasis is indicated via the psoriasis area severity index (PASI) scale, which involves the areas affected as well as the severity of the area affected upon. Women tend to have lower PASI scores in comparison to men. Despite more women being affected by psoriasis, they do not suffer from severe symptoms [20]. It is unclear as to why men suffer from more severe symptoms of psoriasis. Furthermore, current research has uncovered that hormonal changes often plays a significant role in triggering the onset of psoriasis for many who are predisposed to it genetically or may cause flare-ups of those already diagnosed. This is because the skin is affected directly by the endocrine system and severely impacted by the hormonal changes of sex hormones, prolactin, glucocorticoids, epinephrine, thyroid hormone, and insulin, which are seen in greater quantity or solely in women [21]. Women typically experience more hormonal changes than men, as well as often contain greater quantities of many of the hormones which directly act as triggers for psoriasis, lending to a potential theory as to why more women are likely to developed psoriasis than men.

Rheumatoid arthritis and Sjogren's syndrome

Autoimmune conditions such as rheumatoid arthritis and Sjogren’s syndrome are hypothesized to occur more in women due to the hormonal changes women experience. Rheumatoid arthritis is characterized by painful, swollen, stiff joints, often accompanied by fever, fatigue, and weightless. It causes chronic inflammation of various joints due to the synovium of the joints being compromised. Typically, the synovium, which is soft tissue lining the joints and tendons, aids with movement, flexibility, and weight impact. Those affected by rheumatoid arthritis experience thickening of the synovium due to the inflammation, causing deterioration of cartilage and bone of the affected joint. It is hypothesized that women between the ages of 40 and 60 are more likely to develop rheumatoid arthritis compared to men, due to undergoing hormonal changes during menopause. Menopause decreases estrogen and progesterone levels, which is thought to serve as a protective mechanism for bones and joints [22]. Estrogen in larger quantities can decrease inflammation by increasing regulatory cytokines such as interleukin-10 (IL-10) and transforming growth factor-β (TGFB), which is why it is believed to act as a protective mechanism against rheumatoid arthritis and Sjogren’s syndrome [22,23].

Sjogren’s syndrome often causes chronic dry eyes and mouth due to the immune system targeting its own salivary and tear glands, causing difficulty swallowing, dry mouth as well as dry, red eyes. Similar to rheumatoid arthritis, it is theorized that Sjogren’s syndrome is also linked to a drop in estrogen levels, which plays a pivotal part in female immunity. As estrogen decreases during menopause, does its ability to decrease inflammation as effectively, allowing for rheumatoid arthritis to occur. Although women are more likely to suffer from autoimmune conditions, they are also better equipped to overcome infections because of their increased production of antibodies [23]. However, in conditions such as Sjogren’s syndrome, the increased level of antibody production along with the decreased levels of estrogen during menopause in women, leads to a rise of such an autoimmune condition to occur. Decreased estrogen levels nor increased production of antibodies in women alone brings forth an autoimmune condition such as rheumatoid arthritis or Sjogren’s syndrome. 

Despite being a rare condition, systemic sclerosis is four times more likely to occur in women than men. Women are typically diagnosed earlier on in life, specifically around childbearing age and endure symptoms for a longer duration of time. Like psoriasis, women with systemic sclerosis do not experience severe symptoms when compared to men. Systemic sclerosis targets multiple systems throughout the body, specifically affecting the skin, joints, lungs, heart, and kidneys. It causes an increased production of collagen with fibrosis, irregular immune system activation as well as vascular abnormalities. Systemic sclerosis, as with other autoimmune disorders, can be attributed to a defect in X inactivation that results in overexpression of many immune regulatory genes [24]. In an experiment conducted in the University of Pittsburgh regarding estrogen levels and systemic sclerosis, it was found that increased estrogen levels may cause skin thickening and organ fibrosis, hence why women are often diagnosed during childbearing age as estrogen levels increase substantially during pregnancy. Similar to other autoimmune disorders, systemic sclerosis further demonstrates the predominance amongst women due to a lack of X-inactivation.

Role of pregnancy and hormones in the onset of autoimmune diseases

Pregnancy results in an influx of hormonal and bodily changes, with hormonal changes continuing until at least one-year post-pregnancy. Such changes serve as a trigger for the development of autoimmune diseases. There are various physiological changes that occur during pregnancy, such as increased basal metabolic rate, lipid levels, and weight gain. Pregnancy will also induce various changes in the levels of hormones such as estriol, progesterone, and prolactin [25]. The fetus, containing foreign antigens, relies on the mother to serve as its host, resulting in immune changes that tend to cause a suppression of the maternal immune system [26]. This is believed to be carried out in order to prevent rejection of the fetus but leads to a suppressed immune system, which can certainly trigger the onset of autoimmune diseases. Hormonal changes will also occur during the post-partum period leading to an increased incidence of certain autoimmune diseases, such as rheumatoid arthritis. A study found that in the post-partum, there is a significant rise in the incidence of rheumatoid arthritis cases having an incidence rate ratio of 1:7 in the 24 months after delivery [27]. A cohort study done by various doctors published in the Journal of Rheumatology revealed a high risk of developing rheumatoid arthritis in the first-year post-partum [OR = 3.8 (95% CI: 1.5, 9.9)] in comparison with subsequent years (P = 0.004) [28]. The amplified risk of rheumatoid arthritis was at its highest during the first three months [OR = 5.6 (95% CI: 1.8, 17.6)] and had decreased during the following nine months [OR = 2.6 (95% CI: 0.8, 7.9)] [29].

The changes in hormone levels in females going through puberty increases their risk of developing autoimmune diseases. A study was conducted in Taiwan to indicate the vast difference in the likelihood of developing an autoimmune disease such as SLE for girls rather than boys. This epidemiological study had indicated a substantial increase in the prevalence of juvenile SLE amongst Taiwanese girls in comparison to boys who were of the same age [30]. The prevalence of SLE in girls at the age of one was 0.65 per 100,000 children, which increased to 6.7 per 100,000 at age seven and eventually to 34.6 per 100,000 at age fifteen. For boys, the prevalence was almost zero per 100,000 at ages 1 and 7, and to 7.8 per 100,000 at age 15. It is also found that for multiple sclerosis, its pubertal onset is rare, with only 3% to 5% of cases reported for individuals under the age of 18 [31]. After the onset of puberty, there is an increase in incidence with pubertal girls found to be at a greater risk for developing multiple sclerosis than pre-pubertal girls [32]. Such reports suggest that the hormonal changes which occur during pubertal development could be an underlying factor in the gender disparity of autoimmune diseases. Evidence has shown that the changing hormonal climate which occurs during the menopausal transition plays a role in the increased susceptibility of peri- and post-menopausal women to autoimmune diseases due to its effect on inflammatory processes. For instance, in women around 50 years of age, the neutrophil percentage dropped, whereas lymphocyte percentage rose [33], thereby subjecting perimenopausal women to an increased risk of lymphocyte-mediated autoimmune diseases.

Autoimmune diseases such as rheumatoid arthritis and SLE affect women over the age of 40 more frequently. It can be deduced that certain autoimmune conditions are more prevalent given the age and physiological state of the patient. For example, researchers are unsure as to why it has been shown that high levels of estrogen and progesterone are protective for disease activity in rheumatoid arthritis. Therefore, pregnancy would be considered protective against the risk of disease development, due to the increase in estrogen and progesterone levels. On the contrary, menopause and post-partum are often associated with disease worsening due to a drop in estrogen and progesterone levels [34]. There is also a surge of female sex hormones during puberty such as estrogen. High levels of estrogen have been observed in the synovial fluid of patients who are affected by both SLE and rheumatoid arthritis. This is due to the action of aromatase on peripheral tissues. Inflammatory cytokines, such as TNFα, IL-1 and IL-6, which are produced by macrophages will stimulate the action of aromatase which in turn is responsible for the conversion of androgens (dehydroepiandrosterone [DHEA], testosterone, progesterone) into 17-β-estradiol. 17-β-estradiol then acts on immunocompetent cells, thereby activating the macrophages, resulting in a cycle that causes the production of pro-inflammatory cytokine production [35].

## Conclusions

Eighty percent of all individuals affected by autoimmune disorders tend to be women due to variation within the sex chromosomes and hormonal changes. Currently, there are no known cures to autoimmune disorders. Many individuals are predisposed to autoimmune disorders, which are activated by a plethora of triggers such as climate, poor diet/lifestyle choices, lack of exercise, increased levels of stress, and/or lack of adequate sleep. Other triggers such as hormonal changes during puberty and pregnancy for women may often be inevitable. Many predisposed to autoimmune disorders may be able to lessen the severity or prevent the condition from arising, altogether by abstaining from smoking, excessive drinking, instilling a healthy diet/lifestyle, exercising, reducing stress factors, and sleeping adequately. Autoimmune disorders can often be hereditary; thus, it is crucial for individuals who are potentially at risk to be aware of their full family history. Furthermore, those potentially at risk should consult a physician for proper testing and analysis to be conducted to first confirm if they are diagnosed with said autoimmune disorder, and if so how to best decrease the severity via pharmacological intervention and/or lifestyle changes.
